# Multi-cancer Detection Using Pattern Formation in Drying Body Fluids: A Systematic Review and Meta-Analysis of Diagnostic Test Accuracy Studies

**DOI:** 10.1177/15330338251333994

**Published:** 2025-09-01

**Authors:** Maria Olga Kokornaczyk, Marcus Reif, Martin Loef, Harald Walach, Natalia Borisovna Bodrova, Paul Doesburg, João Vitor da Costa Batista, Jürgen Pannek, Devika Shah, Mario Castelàn, Stephan Baumgartner

**Affiliations:** 1 Society for Cancer Research, Arlesheim, Switzerland; 2 27210 University of Bern, Institute of Complementary and Integrative Medicine, Bern, Switzerland; 3 Society for Clinical Research, Berlin, Germany; 4 Change Health Science Institute, Basel, Switzerland; 5 Next Society Institute, Kazimieras Simonavicius University, Vilnius, Lithuania; 6 International Research Group on Very Low Dose and High Dilution Effects (GIRI), Dornach, Switzerland; 7 Institute for Integrative Medicine, University of Witten/Herdecke, Witten, Germany; 8 Federal University of Rio de Janeiro, Multidisciplinary Laboratory of Pharmaceutical Sciences, Faculty of Pharmacy, Rio de Janeiro, Brazil; 9 Neuro-Urology, Swiss Paraplegic Center, Nottwil, Switzerland; 10 Department of Urology, Inselspital, Bern University Hospital, 27210 University of Bern, Bern, Switzerland; 11 Robotics and Advanced Manufacturing, Center for Research and Advanced Studies of the National Polytechnic Institute, Ramos Arizpe, Mexico

**Keywords:** patterns in drying body fluids, diagnostic tests, multi-cancer detection, copper chloride crystallization of blood, droplet evaporation, meta-analysis

## Abstract

**Introduction:**

The ability to detect multiple cancer types with high sensitivity has the potential to reduce diagnostic delays and improve treatment outcomes. Diagnostic patterning tests (DPTs), which utilize self-organized patterns in drying body fluids, are a relatively unexplored diagnostic method. This systematic review and meta-analysis assessed their accuracy for multi-cancer detection.

**Methods:**

Searches were conducted in PubMed, Web of Science, eLibrary Russia, and other databases for studies evaluating DPT accuracy in cancer detection. Study quality was assessed using the QUADAS-2 tool. Data were analyzed for (i) untreated cancers, (ii) treated cancers, and (iii) precancerous conditions, with controls comprising (iv) healthy individuals and (v) non-cancer patients. Meta-analysis adhered to the Cochrane Handbook for Systematic Reviews of Diagnostic Test Accuracy.

**Results:**

Of the 610 identified records, 41 studies involving 15,969 participants were included, encompassing 5265 cancer cases and 189 precancerous condition cases. Pooled sensitivity and specificity across all DPTs were 0.89 (95% CI, 0.83-0.93) and 0.90 (95% CI, 0.84-0.93), respectively. Copper chloride crystallization applied to blood demonstrated the highest sensitivity (0.93; 95% CI, 0.87-0.96) and specificity (0.93; 95% CI, 0.85-0.97), though differences between tests were not statistically significant.

**Conclusion:**

Despite high heterogeneity and the potential risk of bias, DPTs showed a satisfactory degree of accuracy in detecting over 50 cancer types. Further research is needed to evaluate their potential for early cancer detection.

## Introduction

Early cancer diagnosis is crucial for improving clinical outcomes and enhancing the likelihood of cancer remission.^1^ Global cancer incidence is on the rise, with 19.1 million new cases reported worldwide in 2020..^2^ Cancer often progresses asymptomatically for extended periods, leading to late diagnosis in symptomatic or emergency cases.^3^ While common diagnostic tests like imaging and biopsies detect specific cancer types in localized areas, current screening tests focus on individual cancers and are limited to the most prevalent types. Diagnostic and screening tests capable of detecting various cancers with high sensitivity in early stages could significantly improve treatment outcomes, prolong survival, reduce treatment-related complications, enhance the quality of life for patients, and potentially lower treatment costs and complexity.^4,5^

The formation of self-organized structures in drying portions or solutions of various body fluids has intrigued scientists for years and continues to be a significant focus in modern science.^6–11^ Numerous diagnostic tests based on this phenomenon have been developed that operate based on the sensitivity of the characteristics of the emerged structures to even minor variations in the body fluid composition. These tests, referred to as diagnostic patterning tests (DPTs), are relatively simple, time-efficient, and cost-effective, offering potential advantages for diagnostics and other medical applications.

In a previously published mapping review,^6^ various DPTs were described that utilize pattern formation in drying body fluids, with or without reagents, for detecting different diseases and physiological states. A categorization of DPTs based on their experimental protocols was proposed.

The mapping review indicated that many experimental DPTs are primarily aimed at detecting cancer, enabling the identification of multiple cancer types. Furthermore, several studies suggested the potential for diagnosing early stages of cancer and precancerous conditions. Additional promising aspects of DPTs include their capability to (i) monitor the progression of cancer and a patient's response to treatment, (ii) localize cancer in some cases, and (iii) perform multi-target diagnoses (including cancer and other diseases).

Contributions: The present study is the first systematic review and meta-analysis of diagnostic test accuracy studies using DPTs for cancer detection; it aims to assess the sensitivity and specificity of significant subgroups of DPTs. The findings of the present study suggest several promising avenues for future research in the field of DPTs.

This study adhered to the PRISMA-DTA guidelines^12^ for reporting systematic reviews and meta-analyses of diagnostic test accuracy studies.

## Materials and Methods

### Protocol and Registration

The systematic review and meta-analysis were registered under PROSPERO registration number CRD42020187440 on 05/07/2020.

### Data Sources and Searches

Literature was collected through searches in the PubMed and Web of Science databases using combinations of terms describing body fluids, methods of pattern acquisition, target conditions, patterns, and diagnoses: (((“body fluid” OR “corporal fluid” OR biofluid OR “bodily fluid” OR blood OR serum OR plasma OR saliva) AND (evaporate* OR desiccat* OR crystallisation OR crystallization OR facies OR dried OR “wedge dehydration”)) OR (“crystallisation test” OR “crystallization test” OR “Bolen test” OR “copper chloride crystallization” OR “copper chloride crystallization”)) AND (cancer OR neoplasm OR malignant OR tumor OR carcin* OR metastasis OR oncolog*) AND pattern AND diagnos*. The Russian eLibrary was searched using corresponding terms in Russian.

The literature database created for the review “Diagnostic tests based on pattern formation in drying body fluids: a mapping review”^6^ was also consulted for publications related to cancer detection. Additionally, literature was obtained by contacting selected authors and experts in the field, as well as from the Goetheanum library (Dornach, Switzerland) and E. Pfeiffer's Archives (a collection of scanned articles and documents from E. Pfeiffer's research laboratory in Spring Valley, New York, USA).

Furthermore, the “cited by” feature of Google Scholar was used and the reference lists of studies were manually searched to identify additional relevant literature.

The search was conducted in December 2022.

### Study Selection

Articles, books, and book chapters addressing cancer diagnosis in humans were considered. Publications written in English, German, Russian, Italian, French, Polish, and Portuguese were included, with no restrictions on the year of publication. Studies were included only if they focused on patterns as the primary outcome. However, studies that applied desiccation of body fluids to prepare specimens for further physicochemical analysis were excluded.

Furthermore, the inclusion of studies was subjected to the provision of information regarding the sensitivity and specificity of the test or data from which these measures could be calculated. Such data were typically presented as absolute counts of true positives (TP), false positives (FP), true negatives (TN), and false negatives (FN).

### Data Extraction

Data extraction from each publication involved recording the following details into spreadsheets: (i) author(s) and publication year, (ii) country, (iii) study design (case-control or cohort), (iv) method of pattern formation, (v) body fluid analyzed, (vi) reference standard used, (vii) technique for pattern evaluation, (viii) number of patients categorized by treated cancers, untreated cancers, unknown treatment status, and precancerous conditions, (ix) number of controls categorized as healthy donors, non-cancer patients, and donors with conditions known to produce false positive results in the Bolen test, and (x) specific counts of true-positive cancer diagnoses, doubtful test readings, false-negative outcomes for patient groups (point viii), and true-negative and false-positive readings for control groups (point ix).

Data extracted for meta-analyses underwent cross-validation by a second evaluator. Additionally, scores resulting from the quality assessment based on QUADAS-2 criteria (see quality assessment section) were incorporated into the spreadsheets. During the quality check, evaluation also included the assessment of precancerous conditions, which were reviewed and confirmed by evaluators.

### Quality Assessment

The quality assessment of the included experimental studies was independently conducted by two evaluators using the QUADAS-2 tool.^13^ QUADAS-2 comprises four main domains for evaluating study quality: patient selection (representativeness and study design), index test (validity and reliability), reference standard (validity and objectivity), and flow & timing (adherence to study procedures and statistical analysis).

In addition to these domains, a set of predefined signaling questions from^14,15^ was incorporated to further enhance the assessment. Initially, the extended QUADAS-2 tool was tested on three studies, and in two subsequent discussion rounds, the selection and refinement of signaling questions were deliberated upon and finalized. Ultimately, seven signaling questions were chosen to complement the QUADAS-2 domains (Table 1).

**Table 1.** Signaling Questions Added to the Domains 1-4 of the QUADAS-2 Evaluation Tool.

Domain	Question	Answer
1	Were the study groups selected from the same population?	(Yes/No/Unclear)
2	Were the positive and negative results of the index-test defined? Was the index-test reproducible? (Yes/No/Unclear) Were the readers of the index-test trained? (Yes/No/Unclear) Was there an observer variability? (Yes/No/Unclear)	(Yes/No/Unclear) (Yes/No/Unclear) (Yes/No/Unclear) (Yes/No/Unclear)
3	Was the outcome of the reference test influenced by the outcome of the index test?	(Yes/No/Unclear)
4	Were indeterminate results handled properly?	(Yes/No/Unclear)

During the quality evaluation, each question was assigned a score (high, uncertain, or low). If there were discrepancies in the ratings, the evaluators discussed them until a consensus was reached.

### Data Synthesis and Analysis

Data from the 2 × 2 tables for the patient (eg, untreated cancer, treated cancer, treatment status unknown, precancerous conditions) and control (eg, healthy, other diseases than cancer, conditions yielding false positive results) subgroups were analyzed in 11 different data scenarios composed of different combinations of patients and controls (including even one study by Kuczkowski et al (1995)^16^ that was excluded from the primary analysis).

The primary analysis concerned a data scenario most closely resembling a cancer-screening situation, including patients with (i) untreated cancers, (ii) cancers with unknown treatment status, and (iii) precancerous conditions, and the following controls: (i) healthy persons and (ii) patients with diseases other than cancer.

The meta-analysis followed recommendations from the Cochrane Handbook for Systematic Reviews of Diagnostic Test Accuracy.^17^ Test comparisons, individual test evaluations, and the influence of covariates were analyzed separately. Due to the exploratory nature of the analyses, no corrections for multiple testing were applied.

**Figure 1.**
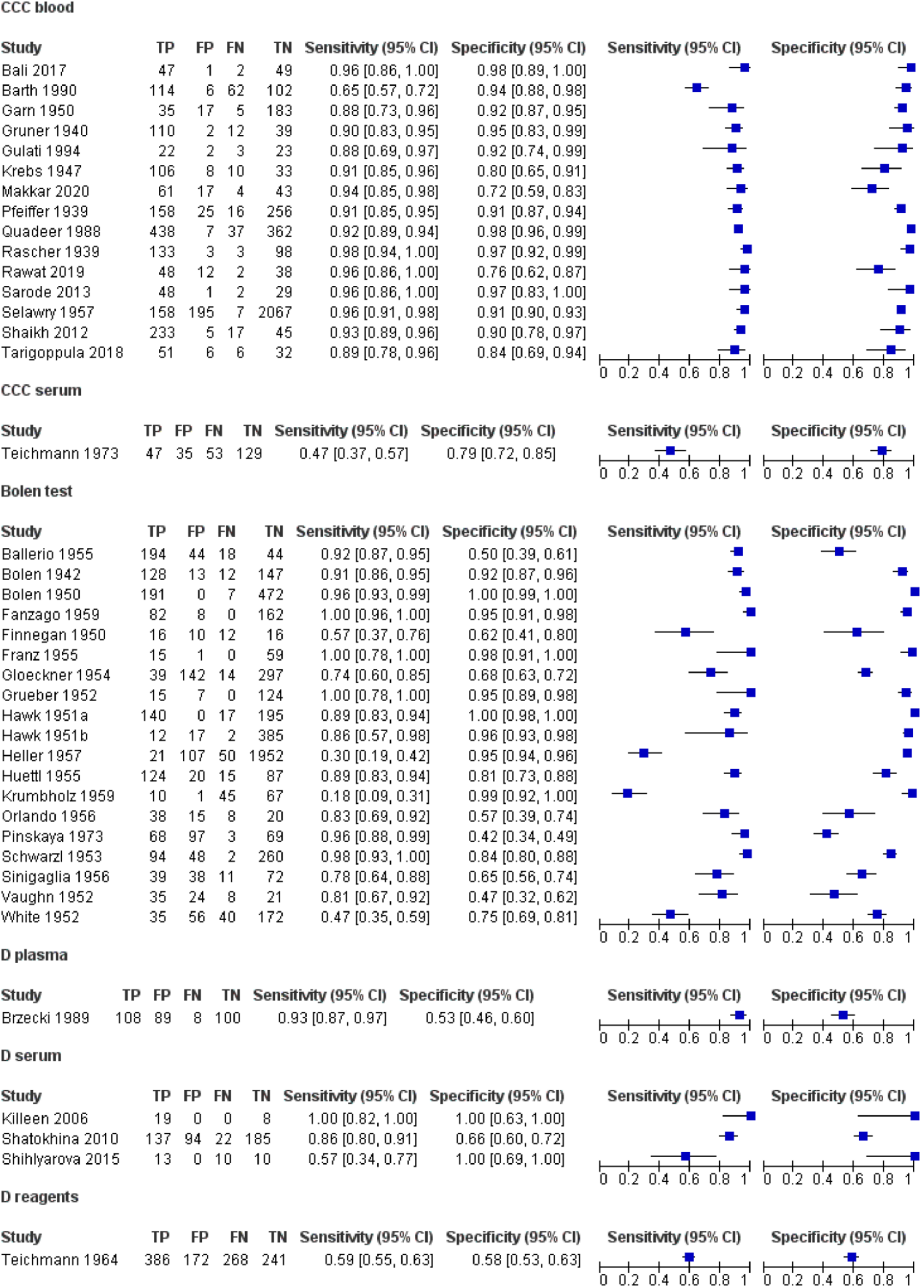
Forest Plot Depicting the 2 × 2 Tables and Sensitivity and Specificity Ranges of the Diagnostic Patterning Tests Applied for the Detection of any Type of Cancer (Data Scenario Resembling a Cancer Screening Setting, ie Exclusion of Treated Cancers and Conditions Yielding False Positive Effects). Legend: CCC – Copper Chloride Crystallization with Additives; D – Droplets; TP – True Positive; FP – False Positive; FN – False Negative; TN – True Negative; CI – Confidence Interval. One Study, Kuczkowski (1995),^13^ has been Excluded from this Analysis due to its Non-Corresponding Patient Sample.

In a first step, exploratory analyses were performed by creating forest plots and summary receiver operating curves (SROC) with the sensitivity and specificity from each study. Subsequently, test accuracy across studies was estimated by fitting hierarchical summary receiver operating characteristic (HSROC) models. For the clinical interpretation, sensitivities with their 95% confidence intervals (95% CI) were derived for the median specificities of the included studies. Additionally, the diagnostic odds ratio (DOR) and the positive and negative likelihood ratio with their corresponding 95% CI were calculated. The HSROC model currently proposed in the Cochrane Handbook assumes equal variances in all groups assessed, so the estimates of sensitivity and specificity and the corresponding CIs resulting from single- and multiple-group analyses can differ. In this article, generally the estimates of the multiple-group analyses were presented, reasoning that the higher sample sizes in these analyses result in more robust variance estimates. To allow an evaluation of this assumption, the results of the primary analyses are presented in Figure 1 as separate single-group estimates and CIs.

The likelihood ratio test was applied to compare the goodness of fit of two or more models that differed with regard to the test or covariates. The statistic is defined as the difference between two times the negative log-likelihood of each model fit (−2LL), which is approximately Chi2 distributed with the number of degrees of freedom equal to the difference in a number of parameters of the compared models.

If multiple groups from a single study were compared and the comparisons were correlated due to shared intervention groups, a potential unit-of-analysis error was addressed in accordance with Chapter 23.3.4 of Higgins et al (2019).^18^

Logistic meta-regression models assessed the heterogeneity and its impact on individual tests.

The analyses were conducted with Revman 5.4 and SAS 9.4, including the MetaDAS macro.^19^

## Results

### Literature Search

**Figure 2.**
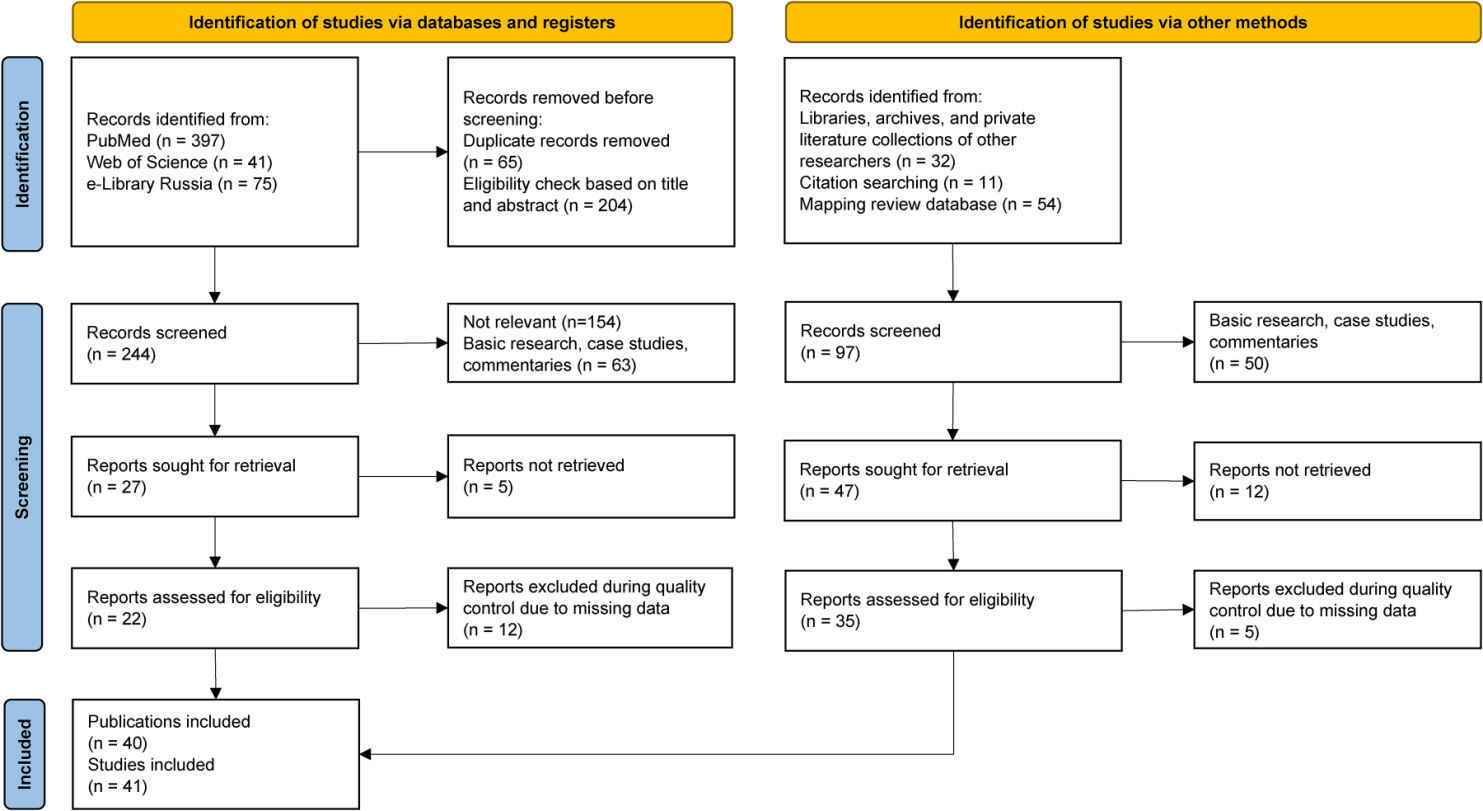
Flow Chart of the Literature Search.

As depicted in Figure 2, a search of the literature databases (PubMed, Web of Science, and eLibrary Russia) using the selected search terms resulted in 513 publications . Additionally, 97 publications were identified through other methods, including searches in libraries, archives, private literature collections of researchers, reference lists, and the database from the mapping review on DPTs.^6^ This brought the total number of identified publications to 610. After removing duplicates, ineligible records, and records that could not be retrieved, 341 publications remained for screening. Of these, 57 publications were deemed eligible for quality assessment. Ultimately, 40 publications met all inclusion criteria and were included in the present systematic review and meta-analysis. Since one publication reported on two studies, a total of 41 studies were analyzed.

### Description of the Literature Included

As shown in Table 2, the majority of the included publications were from the 1940s and 1950s, with 23/40 published between 1939 and 1957. Few publications appeared in the 1960s and 1970s. From 1988 onwards, the number of publications on DPT increased again, with 14/40 published between 1988 and 2021, including seven in the last 10 years. The older studies were primarily published in German-speaking countries and North America, mainly focusing on copper chloride crystallization (CCC) of whole blood or blood droplet desiccation (Bolen test). Recent studies, predominantly published in India and Russia, have focused mainly on the CCC method applied to blood and new approaches based on serum and plasma droplet evaporation.

**Table 2.** Studies Included in the Systematic Review and meta-Analysis.

Author(s) (year)	Study Type	Method / Body Fluid	Reference Standard
Bali and Marathe (2017)^20^	Case-control	CCC / blood	Unclear
Ballerio & Chierego (1955)^35^	Cohort	DD / blood	Histophatology
Barth (1990)^21^	Unclear	CCC / blood	Histophatology and cytology
Bolen (1942)^36^	Cohort	DD / blood	Clinical examination
Bolen (1950)^37^	Cohort	DD / blood	Clinical examination
Brzecki et al (1989)^53^	Cohort	DD / plasma	Clinical examination
Fanzago (1959)^38^	Cohort	DD / blood	Histophatology
Finnegan et al (1950)^38^	Unclear	DD / blood	Unclear
Franz (1955)^40^	Case-control	DD / blood	Unclear
Garn (1950)^22^	Case-control	CCC / blood	Clinical examination
Glöckner (1954)^41^	Cohort	DD / blood	Unclear
Grueber and Huppertz (1952)^42^	Cohort	DD / blood	Unclear
Gruner (1940)^23^	Unclear	CCC / blood	Clinical examinaton
Gulati et al (1994)^24^	Case-control	CCC / blood	Histophatology
Hawk et al (1951) pilot^43^	Case-control	DD / blood	Histophatology
Hawk et al (1951) main^43^	Cohort	DD / blood	Histophatology
Heller (1957)^44^	Cohort	DD / blood	Unclear
Huettl et al (1955)^45^	Unclear	DD / blood	Histophatology
Killeen et al(2006)^54^	Case-control	DD / serum	Unclear
Krebs (1947)^25^	Case-control	CCC / blood	Histophatology
Krumbholz (1959)^46^	Cohort	DD / blood	Unclear
Kuczkowski (1995)^16^	Case-control	CCC / blood	Clinical examination
Makkar et al (2020)^9^	Case-control	CCC / blood	Histophatology
Orlando et al (1956)^47^	Unclear	DD / blood	Biopsy, radiology, histopathology, clinical examination
Pfeiffer & Miley (1939)^26^	Unclear	CCC / blood	Operation, biopsy, post-mortem examination, peritoneoscopy
Pinskaia & Sergeeva (1973)^48^	Unclear	DD / blood	Histophatology
Quadeer (1988)^27^	Case-control	CCC / blood	Unclear
Rascher and Trumpp (1939)^28^	Cohort	CCC / blood	Clinical examination
Rawat et al (2019)^29^	Case-control	CCC / blood	Biopsy
Sarode (2013)^30^	Case-control	CCC / blood	Histophatology
Schwarzl (1953)^49^	Case-control	DD / blood	Unclear
Selawry and Selawry (1957)^31^	Case-control	CCC / blood	Histophatology
Shaikh et al (2012)^32^	Case-control	CCC / blood	Unclear
Shatokhina & Shabalin (2010)^55^	Cohort	DD / serum	Histophatology
Shihlyarova et al (2015)^56^	Unclear	DD / serum	Unclear
Sinigaglia (1956)^50^	Case-control	DD / blood	Histophatology
Tarigoppula (2018)^33^	Case-control	CCC / blood	Clinical examination
Teichmann & Ziebarth (1973)^34^	Case-control	CCC / serum	Unclear
Teichmann (1964)^57^	Cohort	DD / serum with reagents	Unclear
Vaughn et al (1952)^51^	Cohort	DD / blood	Unclear
White et al (1952)^52^	Cohort	DD / blood	Histopathology

DD – droplet desiccation, CCC – copper chloride crystallization.

The included studies either followed a case-control design with well-defined patient and control groups (18/41 studies) or were cohort studies (15/41 studies). Eight studies (8/41) were rated as having an unclear design due to insufficient information.

According to a categorization proposed in,^6^ the included studies applied the following DPTs: CCC applied to whole blood^9,16,20–33^ or serum^34^ desiccation of blood droplets (Bolen test),^35–52^ plasma droplets,^53^ or serum droplets^54–56^; and desiccation of serum droplets with added reagents^57^ (Table 2).

Index tests based on the desiccation of body-fluid droplets involved evaporation-induced pattern formation in body fluids, with or without added reagents, deposited as droplets on a glass surface. In CCC, pattern formation occurred in an aqueous bulk solution (6-10 mL) containing hemolyzed blood or serum and copper chloride dihydrate. Unlike droplet-evaporation-based DPTs, where the body fluid is undiluted, in CCC, the concentration of the body fluid exceeds 1:1000 (v/v).

Depending on the cancer type, the reference standards used included histopathological analyses, clinical examinations, biopsies, or other types of examinations.

In total, the studies involved 5176 cancer patients (967 untreated, 784 treated, and 3425 with unknown treatment status) and 189 patients with precancerous conditions. The control groups included 10,704 individuals, comprising 4599 healthy donors, 5449 non-cancer patients, and 656 cases with conditions known to yield false positive results in the Bolen test (pregnancy, postpartum, post-surgery, blood transfusion, acute tuberculosis; Table 3).

**Table 3.** Studies Included in the meta-Analysis with Specified Numbers of (i) Cancer Patients with Unknown Treatment status (ca); (ii) Untreated Cancers (caUT), (iii) Treated Cancers (caT); and (iv) Precancerous Conditions (pre-ca); and Controls Divided into (i) Healthy (H), (ii) Patients with Other Than Cancer Diseases (OD); and (iii) Conditions Yielding False Positive Outcomes (CFP).

Author(s) (year)	Patients	Controls
Bali and Marathe (2017)^20^	49 pre-ca	50 H
Ballerio & Chierego (1955)^35^	212 ca	88 OD
Barth (1990)^21^	132 ca, 44 pre-ca	108 OD
Bolen (1942)^36^	140 ca	160 OD, 20 CFP
Bolen (1950)^37^	198 caUT, 128 caT	135 H, 337 OD
Brzecki et al (1989)^53^	116 ca	189 OD
Fanzago (1959)^38^	82 caUT, 31 caT	50 H, 120 OD, 120 CFP
Finnegan et al (1950)^39^	28 ca	26 OD
Franz (1955)^40^	15 ca	35 H, 25 OD
Garn (1950)^22^	40 ca	200 H
Glöckner (1954)^41^	53 ca	86 H, 253 OD, 97 CFP
Grueber and Huppertz (1952)^42^	15 ca	18 H, 113 OD, 14 CFP
Gruner (1940)^23^	122 caUT, 5 caT	2 H, 39 OD
Gulati et al (1994)^24^	25 ca	25 H
Hawk et al (1951) pilot^43^	157 ca	195 H
Hawk et al (1951) main^43^	14 ca	7 H, 395 OD, 89 CFP
Heller (1957)^44^	71 ca	107 H, 1952 OD, 262 CFP
Huettl et al (1955)^45^	139 ca	107 OD, 47 CFP
Killeen et al(2006)^54^	19 ca	8 H
Krebs (1947)^25^	116 ca	41 OD
Krumbholz (1959)^46^	55 ca	68 OD
Kuczkowski (1995)^16^	21 caT	10 H
Makkar et al (2020)^9^	65 caUT	60 H
Orlando et al (1956)^47^	46 ca	20 H, 15 OD
Pfeiffer & Miley (1939)^26^	174 ca	281 OD
Pinskaia & Sergeeva (1973)^48^	71 ca	30 H, 136 OD
Quadeer (1988)^27^	475 ca	320 H, 49 OD
Rascher and Trumpp (1939)^28^	136 ca	101 H
Rawat et al (2019)^29^	50 caUT	50 H
Sarode (2013)^30^	50 caUT	30 H
Schwarzl (1953)^49^	96 ca	308 H
Selawry and Selawry (1957)^31^	165 caUT, 599 caT	2262 H
Shaikh et al (2012)^32^	211 ca, 39 pre-ca	10 H, 40 OD
Shatokhina & Shabalin (2010)^55^	132 caUT, 27 pre-ca	25 H, 254 OD
Shihlyarova et al (2015)^56^	23 caUT	10 H
Sinigaglia (1956)^50^	50 ca	100 H, 10 OD
Tarigoppula (2018)^33^	27 caUT, 30 pre-ca	38 H
Teichmann & Ziebarth (1973)^34^	100 ca	130 H, 34 OD
Teichmann (1964)^57^	654 ca	176 H, 237 OD
Vaughn et al (1952)^51^	43 ca	1 H, 44 OD, 7 CFP
White et al (1952)^52^	75 ca	228 OD

### Quality Assessment

**Figure 3.**
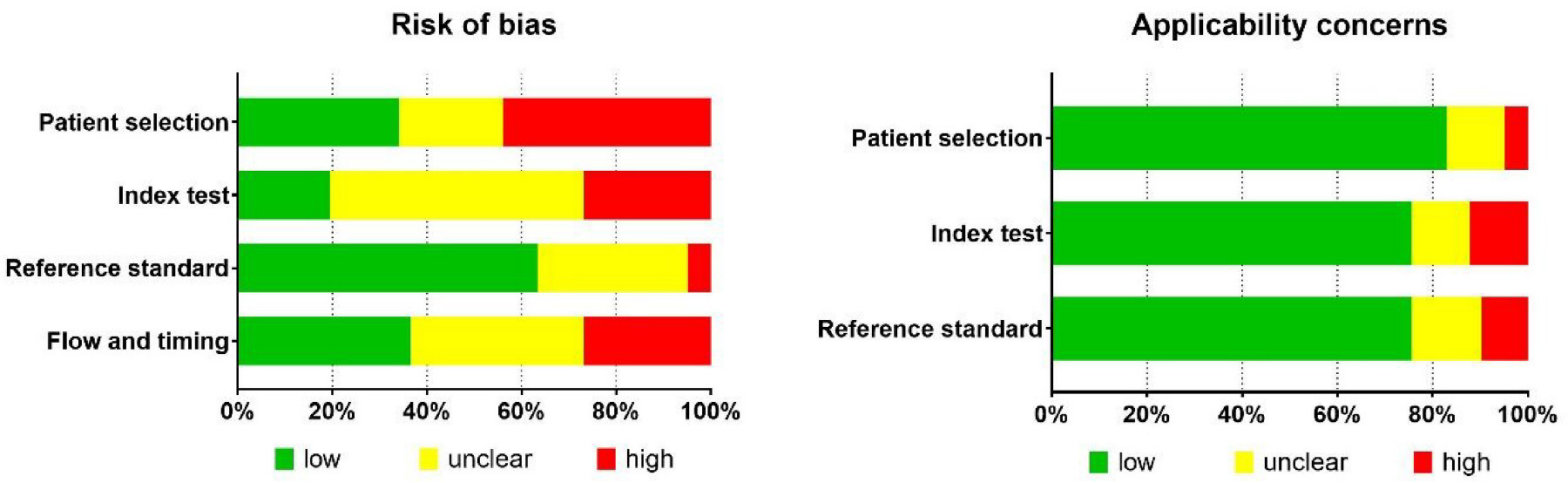
Percentages of Studies that Received Low, Unclear, and High Scores in the Quality Evaluation Following the QUADAS2-tool.

The quality assessment, conducted using the QUADAS-2 tool with additional signaling questions, indicated that the highest risk of bias was associated with patient selection (rated “high” in 18/41 studies), followed by the index test (the investigated test for cancer detection) and flow & timing (each rated “high” in 11/41 studies). The index test and flow & timing criteria also received a high number of unclear ratings (24 and 17/41 studies, respectively) (Figure 3). The reference standard criterion was evaluated as the least likely source of bias, with a “low” risk rating in 28/41 studies. There were only a few studies with applicability concerns related to the index test and reference standard (Table 4).

**Table 4.**
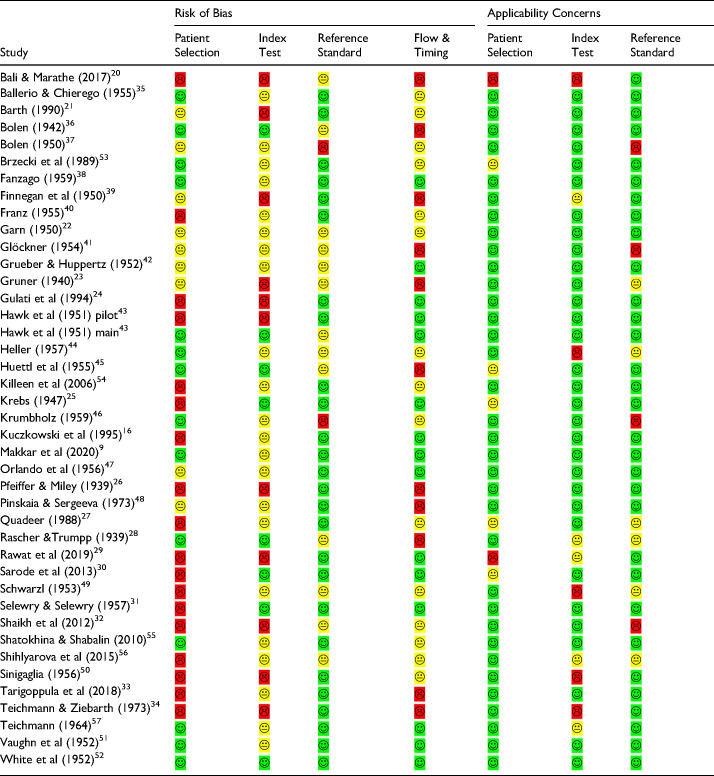
Results of the Quality Assessment of Publications Following the QADAS 2-Tool with Additional Signaling Questions.

### Diagnostic Accuracy of the Patterning Tests

The main outcome of the present analysis are the sensitivity and specificity ranges of the studies, obtained from a data scenario most closely resembling a cancer screening situation (Figure 1). This data scenario included the following patient and control groups: (i) untreated cancers, (ii) cancers with unknown treatment status, (iii) precancerous conditions, (iv) healthy persons, and (v) patients with diseases other than cancer. The scenario excluded (i) patients with treated cancers and (ii) controls with conditions yielding false positive results in the Bolen test, as these conditions would be known a priori in a screening context. Forty out of 41 studies fit this scenario; one study involving only treated cancers was excluded [Kuczkowski (1995); x2 table: TP = 15; FP = 1; FN = 6; TN = 9; sensitivity and specificity (CI 95%) 0.71 (0.48, 0.89) and 0.91 (0.90, 0.93), respectively].

The overall sensitivity and specificity values, indiscriminately pooled over all tests and studies within the scenario resembling cancer screening, were 0.89 (95% CI: 0.83 to 0.93) and 0.90 (95% CI: 0.84 to 0.93), respectively.

**Figure 4.**
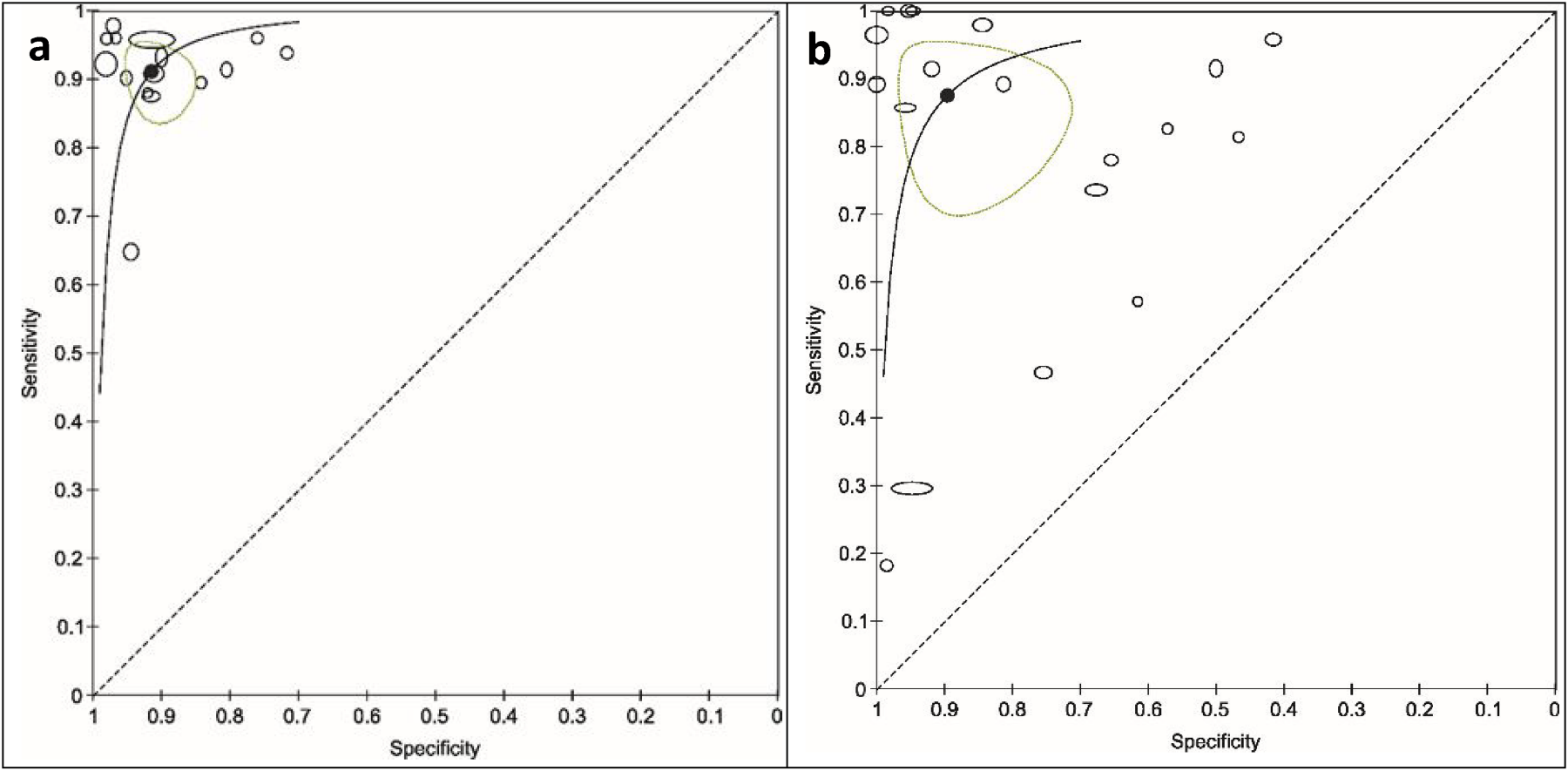
Summary Receiver Operating Characteristic Curves for the Detection of Cancer for the Copper-chloride Crystallization (CCC) of Blood (a) and the Bolen Test (b) with Displayed Individual Study Estimates (Black Ovals; Width and Height of the Study-markers Represent the Relative Number of Subjects on Which the Estimation of Sensitivity and Specificity, Respectively, is Based), Summary Point (Black Dot) and its 95% CI (Green Oval Area Surrounding the Summary Point) and ROC Curves (Black Lines). Parameter values were Estimated Separately for each Diagnostic Test. Sensitivity – Detection of True Positives; Specificity – Detection of True Negatives.

Statistical comparisons between DPTs focused only on CCC applied to blood and the Bolen test (evaporation of blood droplets per se; Figure 4a, b) due to an insufficient number of studies on other DPTs . The sensitivity and specificity for CCC were 0.93 (95% CI: 0.87 to 0.96) and 0.93 (95% CI: 0.85 to 0.97), and for the Bolen test, 0.86 (95% CI: 0.77 to 0.92) and 0.89 (95% CI: 0.80 to 0.94), respectively. However, the −2Log likelihood difference between the two tests was insignificant (chi-square = 2.561; df = 3; p = 0.464).

To obtain secondary outcomes regarding the influence of the selected study groups on the estimated diagnostic test accuracy, the calculations were repeated in modified data scenarios with different population subgroups.

**Figure 5.**
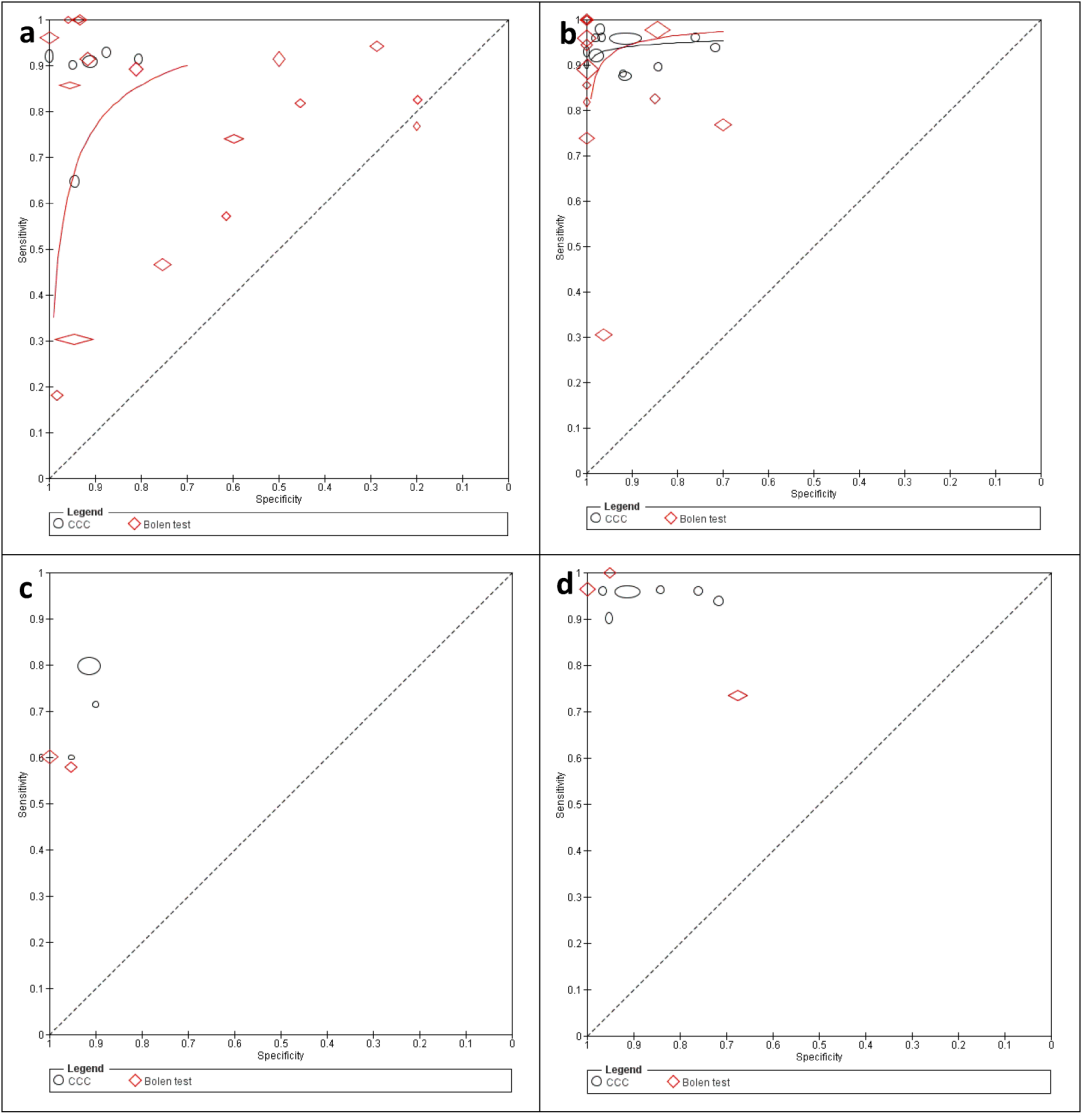
Summary Receiver Operating Characteristic Curves for the Detection of Cancer for the Bolen Test and Copper-chloride Crystallization (CCC) of Blood Following a Data Scenario Including a Common Cancer Patient Group Assessed Against Non-cancer Patients (a) and Healthy Subjects (b) and a Common Control Group Assessed Against Treated-only (c) and Untreated-only Cancer Patients (d). Width and Height of the Study-markers Represent the Relative Number of Subjects on which the Estimation of Sensitivity and Specificity, Respectively, is Based. For (c, d) only Accuracy Values for Individual Studies are Given.

Specifically, the test accuracies were compared (i) in a common group of cancer patients assessed either against healthy subjects or non-cancer patients and (ii) in a common group of controls assessed either against only treated cancer patients or untreated cancer patients (Figure 5).

Regarding (i), the Bolen test showed higher specificity than the CCC when only healthy subjects were included as the non-cancer group (0.99 [95% CI: 0.95 to 1.00] compared to 0.94 [95% CI: 0.84 to 0.98], chi-square = 6.469; df = 3; p = 0.091), while CCC seemed more accurate in discriminating between overall non-cancer and cancer patients (0.95 [95% CI: 0.74 to 0.99] compared to 0.83 [95% CI: 0.67 to 0.92]), although this result was not statistically significant (chi-square = 2.304; df = 3; p = 0.512).

For (ii), the number of studies with patients with known cancer treatment status (untreated or treated) was low for both the Bolen and CCC tests so that this result can be presented only by each study's individual diagnostic accuracy values. The diagnostic accuracy values of studies including untreated rather than treated cancer patients tended to be higher.

Further, for a data scenario including patients with precancerous conditions and a common control group, only four studies concerning CCC were applied to blood. The sensitivities of these studies ranged from 0.50 to 0.95, and the specificities from 0.84 to 0.98.

In another data scenario, patients with pre-existing conditions known to yield false positive results in the Bolen test were excluded. Here, the Bolen test showed enhanced test accuracy; however, this difference was not statistically significant compared to the scenario where these conditions were included.

Finally, the analysis considered also the influence of cases where the diagnostic test reading was doubtful, which applied only to the CCC and Bolen tests. In both tests, excluding the doubtful cases did not affect the sensitivities (0.93 [95% CI: 0.86 to 0.96] and 0.86 [95% CI: 0.76 to 0.92] for CCC and Bolen test, respectively).

**Figure 6.**
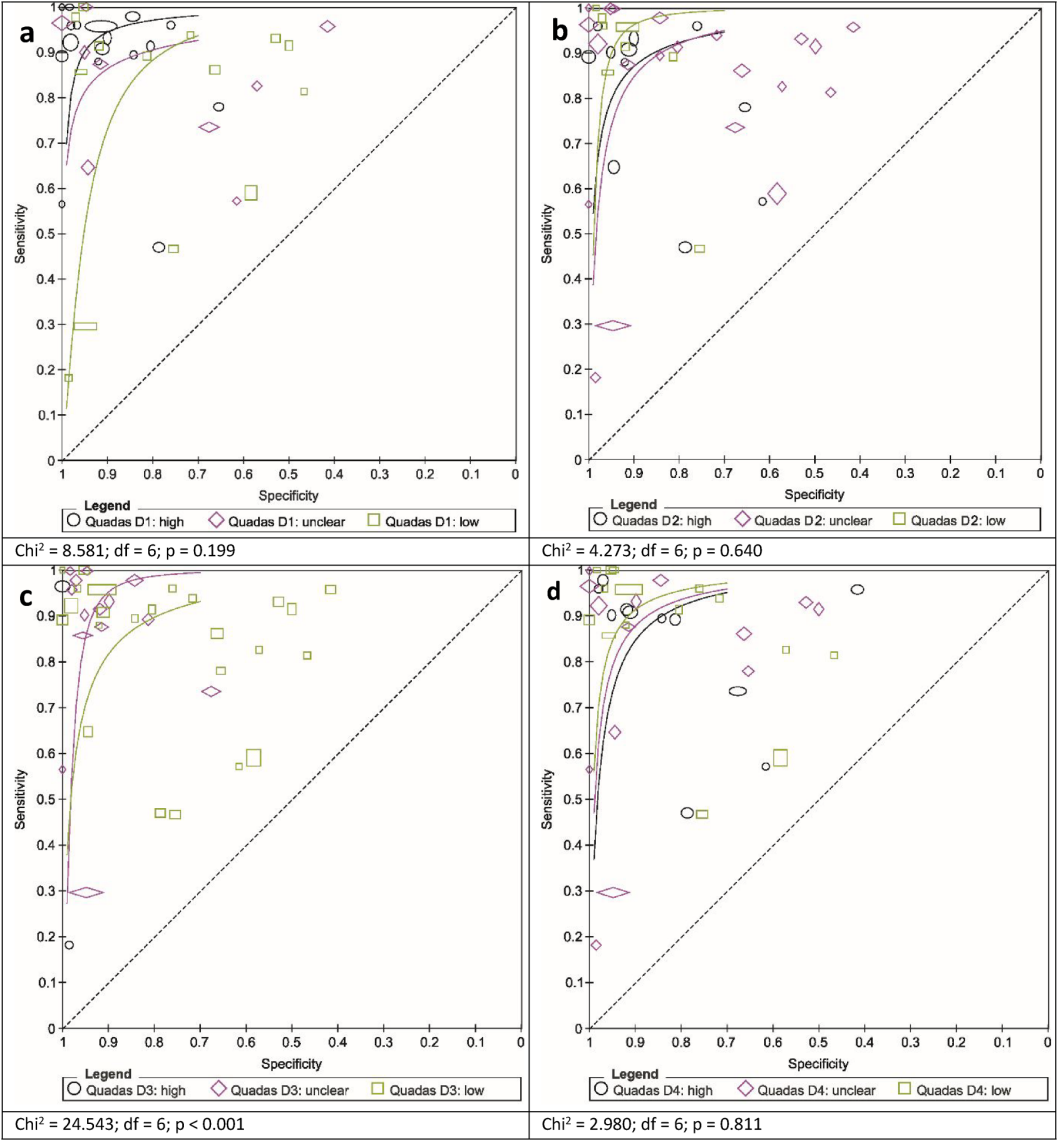
Summary Receiver Operating Characteristic Curves for all Diagnostic Tests with the Four QUADAS 2-tool Domains as Covariates: Patient Selection (a), Index Test (b), Reference Standard (c), and Flow & Timing (d), Named as QUADAS D1-D4, Respectively. Study Points and a Summary Curve are Displayed for n ≥ 10 Studies; Sizes of the Point-markers Indicate the Study-group Sizes.

The influence of study quality on diagnostic test accuracy, as assessed by the four QUADAS-2 domains, was estimated based on all diagnostic tests (Figure 6) . Some tendencies were observed in all domains, reaching significance only for the reference standard domain. In the patient selection and reference standard domains, a higher risk of bias and increased applicability concerns were associated with higher test accuracy, whereas in the index test and flow & timing domains, this association was reversed.

## Discussion

The results show that the sensitivity and specificity across all studies, when pooled indiscriminately over all diagnostic patterning tests (DPTs), amount to 0.89 (95% CI 0.83 to 0.93) and 0.90 (95% CI 0.84 to 0.93), respectively, covering over 50 different cancer types.

Following the proposed classification of DPTs,^6^ the assessed studies applied six different index tests based on the desiccation of (i) blood (Bolen test), (ii) serum, or (iii) plasma droplets per se, and (iv) serum droplets with reagent addition, as well as copper chloride crystallization (CCC) of (v) blood or (vi) serum. The high heterogeneity of the collected publications and the number of studies regarding the distinct index tests only allowed for the comparative analysis of two DPTs (CCC applied to blood and the Bolen test). CCC showed sensitivity and specificity values of 0.93 (95% CI 0.87 to 0.96) and 0.93 (95% CI 0.85 to 0.97), respectively, and the Bolen test showed 0.86 (95% CI 0.77 to 0.92) and 0.89 (95% CI 0.80 to 0.94), respectively. However, there was no significant difference between the two tests (−2LL = 2.561; df = 3; p = 0.464). Both DPTs were more accurate for detecting untreated cancers than those under treatment. The highest specificity was obtained for the Bolen test in a data scenario comparing cancer patients to healthy controls rather than non-cancer patients.

In comparison, recently published meta-analyses of screening test accuracy studies on single-cancer detection reported the following sensitivities and specificities: 0.85 and 0.87 for breast cancer detection using scintimammography^58^; 0.74 and 0.88 for prostate cancer detection using multiparametric MRI^59^; and for colorectal cancer diagnosis using fecal immunochemical testing for hemoglobin, 0.90 and 0.87 in symptomatic patients or 0.69 and 0.87 in a screening population.^60^

Diagnostic test accuracy studies on multi-cancer detection (up to 50 different cancers) reported sensitivities and specificities of: (i) 0.18–0.93 (depending on cancer stage) and 0.99, respectively, for tests based on methylation signatures in cell-free DNA^4,5^; (ii) 0.68–1.00 (depending on cancer type) and 0.99, respectively, for a blood diagnostic model based on microRNA^61^; and (iii) 0.62 (for stage I disease) and 0.95, respectively, for cancer detection using free glycosaminoglycans in plasma and urine (also allowing the prediction of a putative cancer location with 89% accuracy).^62^

The application of the QUADAS-2 tool for the assessment of risk of bias and applicability (Figure 3, Table 4) indicated a high risk of bias in the included studies. The highest risk of bias was associated with the domain patient selection (rated high in 43.9% of the studies), primarily due to the inclusion of patient and control groups with predefined conditions (case-control design). Further, the risk of bias in the domain index test was also notably high (rated as high in 26.8% and unclear in 53.7% of the studies). In DPTs, the test reading consists of evaluating the presence or intensity of certain pattern features. For cancer, in CCC, the evaluated feature is a so-called transverse formation (ie, needles or branches running perpendicular to the radially arranged crystal needles), while in the Bolen test, it is the incoherence of the structural clusters formed in the inner zone of the blood droplet. These cancer features for the two tests were consistent across all studies. Despite the well-defined positive and negative test results, the threshold was often undefined, or there was a lack of information on the index test reproducibility or the readers’ training.

The application of the QUADAS-2 tool for assessing risk of bias and applicability does not fully address several important quality requirements for diagnostic test accuracy studies. These requirements include batch effects, population biases related to age and lifestyle, completely separate training and validation cohorts, randomization of cases and controls, and perfect observer blinding. Additionally, most of the studies included in this review are old (published before the QUADAS-2 criteria were established), which further limits the applicability of the quality assessment.

Further development of the DPT methodologies related to the control of the evaporation environment^63^ and the implementation of modern image evaluation and recognition tools (eg, deep learning and machine learning) might reduce the risk of bias associated with index tests and help to fully exploit these tests’ diagnostic potential.

Deep learning can be utilized to extract intricate features from the patterns formed in desiccated body fluids, while traditional machine learning methods, such as support vector machines (SVM), can be used to classify these features. A hybrid approach of this nature has been previously demonstrated by some authors of this paper in the analysis of dried droplets of aqueous plant-based extracts.^64–67^ Further inspiration can be drawn from successful implementations in other fields, such as malware detection,^68^ improving reproducibility, and minimizing biases associated with manual pattern interpretation.

Another critical consideration when using AI to analyze self-organized patterns in body fluids is the time complexity of the models. Computational efficiency and scalability are essential, particularly in clinical applications where systems must process data swiftly and reliably. Future research should evaluate the time and resources required by these models to ensure their feasibility for integration into clinical environments. This need for rapid and efficient processing is closely tied to data security, as adversarial attacks could manipulate input data (eg, pattern images), leading to erroneous results and compromising diagnostic reliability. Adversarial training^69^ which involves using intentionally manipulated data to enhance model robustness, presents a promising strategy to mitigate these threats. As pointed out in,^70^ it is important to explore methods that strengthen models against malicious interference, thereby ensuring their safe and effective application in clinical settings. Finally, a balance between diagnostic accuracy and operational practicality is essential to ensure that AI-based tools can be seamlessly and securely integrated into medical diagnostic systems.

Some features of the diagnostic tests are worth mentioning. CCC was the only test capable of detecting precancerous conditions^20,21,32,33^ and identifying the cancer site by evaluating the transverse formation's position relative to the crystallization pattern's center.^31,32^ The Bolen test was the simplest, quickest, and least expensive, but since it is designed for capillary whole blood analysis without anticoagulants, it could only be performed at the patient's bedside. This likely explains why the Bolen test was primarily studied in the past (1942-1973). In contrast, recent publications (2006-2015) regarding droplet evaporation of undiluted body fluids have used serum or plasma instead of blood, as these can be frozen and, therefore, easier to handle.

Reassuming, the limitations of the present study are, above all, the high heterogeneity of the collected studies as well as the risk of bias related to the case-control design and index test threshold. The results of the included diagnostic test accuracy studies might also be biased by the evaporation of body fluids or body fluid solutions at room conditions (eg unprecise control of temperature and relative humidity) and by the mostly non-standardized, visually performed pattern evaluation.

It should be emphasized that the purpose of this systematic review and meta-analysis is purely exploratory, aiming solely to identify promising research directions for future systematic studies on DPTs, rather than to propose DPTs as diagnostic tests for cancer.

During the completion phase of the present systematic review and meta-analysis, the authors of the present study learned that Sigmund Rascher, the first author of one of the publications included here (Rascher and Trumpp, 1939),^28^ had carried out human experiments on inmates of the Buchenwald and Dachau concentration camps in 1942 and 1943.^71^ The study included in the present analysis was published some years before these criminal experiments were carried out. Based on purely scientific reasoning, one has to adhere to the study protocol published, where no exclusion criteria had been defined for the unethical behavior of the study authors, and therefore, the main analysis presented is based on all publications, including Rascher and Trumpp (1939). However, for ethical reasons, a sensitivity analysis excluding this publication was also performed. The results of this sensitivity analysis were within the confidence intervals of the main analysis: sensitivity and specificity of DPTs across all tests and studies after exclusion of Rascher and Trumpp (1939): 0.88 (CI: 0.83-0.92) and 0.89 (CI: 0.83-0.93), respectively, and for CCC: 0.92 (CI: 0.88-0.94) and 0.91 (CI: 0.87-0.94), respectively. Thus, neither the inclusion nor exclusion of Rascher and Trumpp (1939)^28^ significantly changes the results of the present analysis.

The use of DPTs for cancer diagnosis was recently addressed in two newly published diagnostic test accuracy studies. These studies, focusing on the diagnosis of bladder^72^ and oral cancer,^73^ respectively, were published after the conclusion of data analysis for the present study and were therefore not included in the meta-analysis. The first study utilized artificial intelligence to evaluate droplet patterns in urine samples with various reagent additions, as well as EDTA-preserved blood samples, reporting sensitivities ranging from 0.68 to 0.99 and specificities ranging from 0.83 to 0.97, depending on the sample type.^72^ The second study employed CCC on peripheral blood samples, achieving a sensitivity of 0.80 and a specificity of 1.00.^73^

## Conclusions

The accuracy of DPTs falls within the range of other current cancer detection tests, indicating that it is sufficiently high to justify further investigation. Targeted validation studies are recommended to explore the potential of these relatively simple methods. In particular, studies should focus on evaluating the accuracy of DPTs for various cancer types, especially those that are challenging to diagnose or not included in population-wide screening programs.^74^ Additionally, investigations should assess their effectiveness for detecting precancerous conditions and different stages of cancer.
